# Plyometric Performance in Under-10 Soccer Players: Effects of Modified Competitions and Maturational Status

**DOI:** 10.3390/s26010068

**Published:** 2025-12-22

**Authors:** Francisco Javier García-Angulo, Antonio García-Angulo, Ricardo André Birrento-Aguiar, Enrique Ortega-Toro

**Affiliations:** 1 Faculty of Sports Science, University of Murcia, 30700 Santiago de la Ribera, Spain; franciscojavier.garcia19@um.es (F.J.G.-A.); ra.birrentoaguiar@um.es (R.A.B.-A.); eortega@um.es (E.O.-T.); 2 Human Movement and Sports Science (HUMSE), Faculty of Sports Sciences, University of Murcia, 30720 Santiago de la Ribera, Spain; 3 Sports Performance Analysis Association (SPAA), Faculty of Sports Sciences, University of Murcia, 30720 Santiago de la Ribera, Spain

**Keywords:** biological maturation, plyometric performance, youth soccer

## Abstract

The aim of this study was to examine the effects of different competition formats on the plyometric performance of under-10 soccer players, while analysing the influence of maturational status. A quasi-experimental design was applied, involving 50 players (mean age = 9.47 ± 0.54 years). Kinematic load was recorded using Wimu™ inertial accelerometers, and maturational status was evaluated using the percentage of predicted adult height (%PAH) as a moderating factor. Results indicate that while total impact volume did not show significant differences (*p* = 0.082), the modified format (MD1) showed a reduction in biomechanical intensity per action. Very large differences were reported in mean take-off acceleration (*p* = 0.001; BF_10_ = 23.97) and landing acceleration (*p* < *0*.001; BF_10_ = 70.57). Furthermore, biological maturation was found to be an essential moderating factor, with a threshold of significance identified at 75.5% %PAH. The results of this study show that modified rules can be a good tool for modulating plyometric intensity without compromising the volume of exposure to the stimulus. These findings may highlight indicate the need to implement an individualized approach, based on maturational thresholds, to optimise adaptations and ensure joint safety during long-term development.

## 1. Introduction

The process of long-term athletic development in youth soccer involves complex interactions between growth, maturation, and training adaptation. During preadolescence, rapid biological and neuromuscular changes occur that significantly influence motor control, coordination, and physical performance [1,2]. This period is characterized by accelerated somatic growth, alterations in body composition, and progressive increases in muscle mass and strength, all of which affect the execution of sport-specific skills [3]. Furthermore, marked inter-individual variability in maturation status means that players of the same chronological age may differ considerably in anthropometric, physiological, and neuromuscular characteristics [4]. Such heterogeneity challenges coaches and practitioners, as uniform training programs or competition formats may not adequately accommodate players at different stages of biological development, potentially constraining skill acquisition or predisposing some athletes to a higher risk of injury [5].

For this reason, studies analyzing the effect of biological maturational have gained particular relevance in recent years [6,7,8]. Most of these studies classify players according to three categorical estimation models: late maturers, early maturers, or players at the appropriate time [9]. However, this grouping presents some methodological limitations. Therefore, some studies are beginning to use maturational age as a continuous variable, allowing for the establishment of more precise effect thresholds [10]. Similarly, within the field of maturational age studies, bio-banding studies have emerged, advocating for competitions based on maturational age rather than chronological age, as this type of competition allows players to compete on a level playing field, among other advantages [11,12]. In this context, it is possible to adjust for differences in explosive and high-intensity actions [13,14,15].

Explosive actions such as sprinting, jumping, and rapid changes in direction are central to successful performance in soccer [14]. These abilities depend on the efficiency of the stretch–shortening cycle, rapid force production, and coordinated motor patterns that allow players to perform repeated high-intensity movements [16,17]. Plyometric activities enhance neuromuscular efficiency and the ability to tolerate impact forces, which are essential for both performance and injury prevention in young athletes [18]. This assertion is reinforced by recent evidence demonstrating how optimising landing strategies can reduce the risk of lower limb injuries. Thus, some studies show that adjusting biomechanical parameters such as the initial contact angle of the ankle and its range of motion during single-leg landings can significantly decrease vertical impact forces and the load transmitted to vulnerable structures such as the anterior cruciate ligament, reducing the risk of injury [19]. Although extensive research has examined the effects of structured plyometric training programs, less attention has been given to the natural neuromuscular stimuli provided by competitive play [20].

Importantly, plyometric actions performed in competition differ in developmental meaning from those performed in training. While training tasks often involve controlled, predictable, and coach-designed stimuli, competitive matches generate plyometric actions under conditions of uncertainty, opposition pressure, time constraints, and tactical decision-making. These contextual demands can amplify the neuromuscular load, modify landing and take-off mechanics, and increase the frequency of unplanned explosive movements, providing a type of stimulus that is difficult to replicate in training. Thus, understanding plyometric responses in real competitive settings is essential for appreciating the full spectrum of neuromuscular development in youth players.

Youth soccer matches are characterized by intermittent and multidirectional movement patterns involving frequent accelerations, decelerations, and changes in direction, as well as jumping and landing actions [21]. The competition format—defined by variables such as pitch size, match duration, and number of players—can substantially influence the type, frequency, and intensity of these actions, thereby determining the overall external and internal load experienced by young players [22]. For instance, reducing field dimensions or the number of participants generally increases ball contact frequency, promotes more high-intensity efforts, and enhances tactical involvement [23].

Despite the importance of understanding how the competitive format influences physical and neuromuscular demands, the available literature on competition is still limited. Most research has focused on analyzing plyometric responses in training situations, through small-sided games or other traditional tasks [24,25,26]. However, there is a lack of studies examining official or tournament-based competitive contexts, where the physiological, tactical, and contextual demands differ substantially from training [26,27]. In addition, within the existing studies, few have specifically addressed youth developmental soccer at early ages, which creates a need to conduct studies in early stages. Furthermore, within the studies on young players, the importance of biological maturation has not been taken into account.

Biological maturation also directly shapes the biomechanical response to these environments. As players progress through maturational thresholds, changes in limb length, muscle architecture, tendon stiffness, and neuromuscular control influence how they absorb and produce force during explosive actions. Early-maturing players typically outperform their peers in measures of strength, speed, and power, leading to more frequent and more effective execution of plyometric actions during competition [3,4]. Consequently, maturation status should be a central consideration when interpreting performance outcomes or designing competition structures, as it affects both exposure to physical stimuli and the trajectory of technical and tactical development [5].

Recent frameworks in youth sport pedagogy have emphasized the importance of designing developmentally appropriate environments that balance physical challenge and skill progression [22,23]. In soccer, adjusting competitive parameters—such as match duration, pitch dimensions, or tournament structures—has been proposed as a practical strategy to optimize player engagement, promote higher physical and technical involvement, and ensure equitable developmental opportunities across maturation stages.

Therefore, the aim of this study was to analyze plyometric responses and high-intensity performance across between two distinct tournament formats in under-10 male soccer players.

## 2. Materials and Methods

A quasi-experimental A-B study was designed, where A corresponded to football tournament with the official FFRM (ST) rules and Condition B corresponded to a tournament with modified rules (MD1).

The initial sample for the study consisted of 51 players. Due to experimental dropout (*n = 1*), the sample studied consisted of 50 U-10 football players with a mean age of 9.47 ± 0.54 years. The players trained twice a week (180 min per week) and played one match. The study was approved by the Ethics Committee of the University of Murcia (ACTA4/2024/CEI).

The independent variable analysed was the competition rule set (Table 1). Thus, two tournaments were held with the following common characteristics: number of players 8 vs. 8; playing area 58 × 38 m; goal size 6 × 2 m. The rules differences between tournaments are presented in Table 1.

The dependent variables analysed were impacts, horizontal impacts, jumps, steps, landings and take-offs. All these variables were analysed in terms of number and g-force, except for steps. The thresholds used for the analysis were for impacts; horizontal impacts; landing and takeoff were: (1) 0–3 g; (2) 3–5 g; (3) 5–8 g; (4) +8 g. For maximum takeoff and maximum landing, cut-off points of 5 g or more were used. These variables were recorded using a Wimu^TM^ inertial device (Realtrack Systems, Almeria, Spain). This device contains, among other types of sensors, four accelerometers that allow the calculation of these types of variables. The data were collected on this device and extracted using SPRO software version 1.00.989.

Before the first tournament, legal guardians were informed of the study’s objectives and signed the informed consent form. At this time, the information necessary for calculating the age of maturity was collected using the Khamis–Roche formula [28]. The above variables were collected in two different tournaments.

Regarding the procedure, the participating teams were contacted and informed about the study’s objectives. Once the teams gave their consent, their legal guardians were contacted and invited to a meeting. At the meeting, they were informed about the research (objectives, duration, protocols, measurement variables, etc.) and the necessary data for calculating maturation age was collected.

Data collection began one week later. Two tournaments were held. These tournaments took place on different days with a three-day interval between them, allowing for favorable weather conditions and time for the players to recover. The matches were played indoors with an average temperature of 17–19 °C in the first tournament and 14–17 °C in the second tournament. The first tournament was played using standard rules (ST), and the second using modified rules (MD1). Each tournament consisted of six matches, with each team participating in three. Before each match, the Wimu devices were attached to the players and removed at the end of the match. To reduce fatigue, charge the devices, and verify their functionality, a minimum 15-min break was established between each match. In addition to this 15-min break, each team had 15 min to warm up. The warm-ups included joint mobility and dynamic stretching, close-range shots without positioning, shots without opposition, and reduced opposition situations.

After each tournament, the data was extracted from the Wimu^TM^ devices and stored on an external hard drive. This data was encrypted, so the researcher processing and analyzing it did not know which player, team, or tournament the information corresponded to.

The data was extracted using Spro software. With this software, the effective playing time was selected, cut-off points were established for some variables. For data filtering, actions during game time were selected and the values during this period were observed. The software used automatically eliminated outlier values, but the researchers checked that there were no particularly anomalous values. The data matrix was created in Excel. In the Excel data matrix, each row represented an action performed by the players. Therefore, it was necessary to organize the matrix so that each row represented a player, and the columns represented the variables studied and both tournaments (repeated measures). The effective playing time was determined by the time during which the game clock/possession clock was running.

### Statistics

The normality of the variables was calculated using the Shapiro–Wilk test. To calculate the differences between tournaments, Student’s *t*-test was used for normal variables and the Wilcoxon test for non-normal variables. In addition, the effect size was calculated using Cohen’s d for normal variables and the biserial rank correlation for non-normal variables. A Bayesian analysis was also performed, which should be considered the primary analysis. This should be interpreted using the following scale: <1/100 support of extreme evidence H0, 1/100 to <1/30 support of very strong evidence H0, 1/30 to <1/10 support of strong evidence H0, 1/10 to <1/3 support of moderate evidence H0, 1/10 to <1/3 support of moderate evidence H0, 1/3 to <1 support of ambiguous evidence H0, 1 to 3 support of anecdotal evidence H1, >3 to 10 support of moderate evidence, >10 to 30 support of strong evidence H1, >30 to 100 support of very strong evidence H1, and >100 support of extreme evidence H1

A moderator analysis was conducted to examine how maturational age (continuous variable) affects the effect of norm modification (differences between ST and MD1). The relationships between the predictor (ST) and outcome (MD1) were examined by testing the interactions between these variables and the stable moderator (W1) using a simple moderation model [28]. Thus, an ordinary least squares regression analysis was conducted using SPSS macro MEMORE v2.1 [28]. The Johnson–Neyman procedure was used to identify areas of significance where the intervention was significant according to maturational age [29]. To assess potential non-linearities, we included quadratic terms of the moderator variable (M^2^) in a parallel model. The terms were found to be non-significant, suggesting that the relationship can be modeled linearly.

## 3. Results

Firstly, the differences between tournaments in the variables associated with the impact-related actions were analysed.

Table 2 summarises the differences between tournaments in terms of the impacts produced. No significant differences were found for any of the variables analysed. The moderator analysis did not find any significant transition point where the effect of the intervention was moderated by maturational age.

The analysis of jumps is presented in Table 3. This table shows the variables of number of jumps, number of steps, take-off forces and landing forces.

Table 3 shows the differences between tournaments in the variables related to jumps. Significant differences were found in Average take-off (g) (t (49) = 3.44; *p* = *0*.001; BF_10_ = 23.97) Average landing (g) (t (49) = 3.83; *p* ≤ 0.001; BF_10_ = 70.57), reporting a very strong Bayesian factor in favour of the alternative hypothesis.

**Figure 1:**
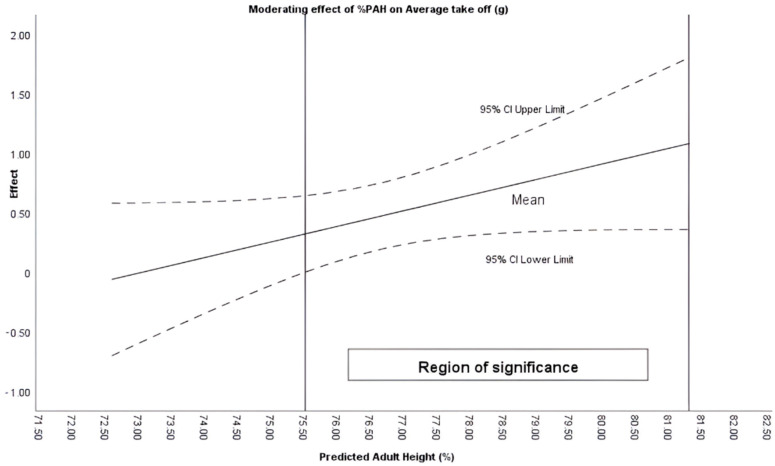
Moderating effect of %PAH on average take off (g).

**Figure 2:**
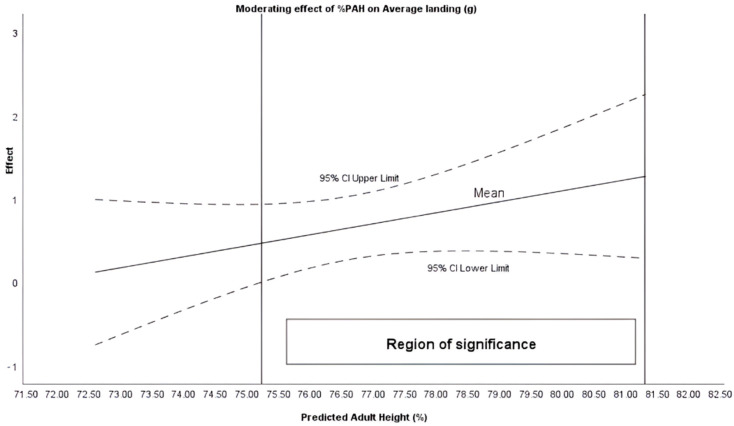
Moderating effect of %PAH on average landing (g).

**Figure 3:**
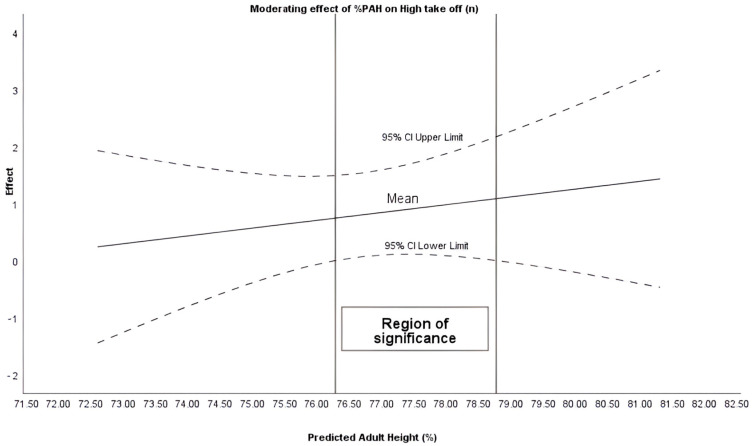
Moderating effect of %PAH on high take off (n).

The Johnson–Newman procedure identified that %PAH moderated the intervention in the variables of Average take-off (when %PAH + 75.51%), Average landing (%PAH + 75.23%) and High take-off (%PAH between 76.28 and 78.77%) (Figure 1, Figure 2 and Figure 3).

## 4. Discussion

The aim of this study was to analyze the impact of two different competition formats, in which the structural variables (playing area and number of players) were kept constant while the internal game rules were modified, on the plyometric and high-intensity performance of U-10 soccer players.

The results of this study suggest the modified internal rules in both competition formats did not produce significative changes in plyometric performance in terms of the total frequency of impacts. This apparent stability in the volume of explosive actions may be relevant to ensure the continuous exposure required for neuromuscular adaptations such as improved power and speed [10,24].

However, this stability in volume does not necessarily imply identical biomechanical demand. A detailed analysis of accelerometry, focused on intensity per action, revealed significant differences in average take-off and landing acceleration (g) between formats. This modulation of intensity suggests that the modified format could induce a distinct biomechanical pattern, potentially altering the quality of the applied stimulus rather than its quantity [18,30,31].

A potentially important finding is the moderating effect of maturational age (%PAH) on the plyometric response. Maturational age appeared to moderate moderates the intervention average take-off, average landing, and maximum jump. These findings are in line with previous literature emphasizing the need to control maturation for proper load management [20,32].

Accordingly, players who are more advanced in maturation tended to exhibit greater force-related magnitudes under the more demanding format. This finding suggests that rules modifications must be individualized and monitored tools—a fundamental principle for safe athletic development [10,24]. The results of this study differ from those reported those found in others, such as other authors in U-13 basketball players, where no moderating effect of maturation was found on certain kinematic variables. Such discrepancies may be explained by differences in sport-specific demands, maturational stage, or the kinematic variables analyzed [32].

The rationale for these modifications extends beyond the neuromuscular domain. The fact that the results of this study do not show negative effects on total plyometric volume allows for this strategic balance. Controlling the stress related to plyometric volume, technical and tactical development can be facilitated and physiological variables of internal load could also be influenced [31,33].

Similarly, the results align with other studies which, analyzing physiological variables, have demonstrated that adapted rules can contribute to healthier sports practice [33]. These findings are consistent with other studies that support the notion that reducing space and the number of players increases heart rate in high-intensity zones [34]. Therefore, modulating biomechanical intensity without compromising plyometric volume may suggest that adapted rules enable specific cardiorespiratory adaptation [35] while potentially preserving joint safety. Furthermore, manipulating operational variables such as periods or scoring could lead to an increase in the density of technical actions and the frequency of decision-making per player. This scaling facilitates skill acquisition and improved cooperation [35,36,37]. Although the results shown identify 75.5% of PAH as a significant threshold, it should be noted that the biological and neuromuscular mechanisms that could explain this critical point have not been fully described. Likewise, it is possible that above this threshold, variables such as musculotendinous stiffness, intermuscular coordination, and force absorption capacity are modified, triggering more demanding responses for the neuromechanical system. During this stage, there are rapid increases in body mass and muscle force generation capacity, which often precede or are out of balance with tendon adaptations, causing greater tensions and fluctuations in tendon deformation [38].

These changes in the properties of the muscle–tendon complex are associated with increases in tendon stiffness with maturation and training, which modifies the behavior of the stretch–shortening cycle and the way forces are absorbed and transmitted during landings and decelerations [39]. In addition, neuromuscular development during adolescence involves a reorganization of intermuscular coordination and the ability to attenuate impact forces; this can result in different landing force–time profiles between groups of different maturity, and a transient increase in vulnerability to load peaks [40]. Therefore, 75.5% PAH could be interpreted as a functional threshold that likely interacts with growth and maturation processes.

Despite these findings, several limitations should be acknowledged. The main limitation lies in quantifying load through relative acceleration (g) instead of Ground Reaction Force (N). Without validation against a force platform (the gold standard), the estimation of applied and absorbed forces is indirect, limiting the ability to translate results into absolute force values. Another limitation is the lack of direct and simultaneous data on physiological (Heart Rate) and technical–tactical variables, which prevents a holistic analysis of compensations between domains. Additionally, the characteristics of the sample are a limitation themselves, as they focus on a very specific age and competitive level. The ICP model may be promising, although these results should be interpreted with caution and corroborated by other studies given the initial exploratory nature of this paper.

The findings carry direct implications for youth football practice. The modified competition format (MD1) can be proposed as a precise load-periodization tool. Coaches can effectively modulate the biomechanical intensity (jump take-off and landing g-forces) while maintaining the total volume of plyometric actions. However, the response to these rule modifications is inherently individualized. Since biological maturation (assessed by %PAH) moderates the intervention effect, coaches should adopt an Individualized Competition Periodization (ICP) approach. Specifically, the maturational threshold of 75.5% Predicted Adult Height (%PAH) serves as a key decision-making point. This threshold should guide the implementation of competition formats that elicit higher biomechanical intensity. Such an evidence-based approach optimizes physiological adaptations and favors joint safety in long-term athletic development.

Future research should explore the longitudinal application of these results. Load modulation through game format invites investigation into the long-term effects of an Individualized Competition Periodization Program. This program should vary competition format according to the maturation thresholds (%PAH) identified in this and other studies, with the goal of optimizing physical and cognitive adaptations. It is essential to extend the study to other competitive levels and ages, establishing a link between biomechanical and physiological responses throughout the athletic development process. Other possible research lines include the need to establish robust regression models for accurately converting wearable acceleration (g) to Newtonian force (N) through force platforms, increasing monitoring validity; and the development of data fusion algorithms integrating biomechanical, spatial (GPS/LPS), and physiological (HR) variables for a three-dimensional and automated load profile. The current technological limitations have not allowed us to accurately distinguish all possible types of plyometric actions. In this regard, future lines of research should differentiate between subtypes of plyometric actions to establish a relationship between plyometric subtypes and maturational age or the effect of rules changes.

## 5. Conclusions

The results appear to confirm that changes in youth football rules are complex and multifaceted. Their value may lie not only in increasing the overall workload, but also in modulating biomechanical intensity and potentially supporting the comprehensive development of players. All of this is in line with a multidisciplinary view of sport. This study seems to indicate that, although the total volume of plyometric impacts remained stable, the intensity per action (average acceleration in g) varied significantly between the different formats, which may support the ability of the portable sensor to discriminate the quality of the stimulus. In addition, biological maturation (%PAH) was identified as an essential moderating factor, indicating that the response to load is intrinsically individualised. It is therefore concluded that adapted rules can be an effective tool for coaches, as they allow physiological adaptation to be optimised without excessive joint overload. The results support the need to evolve towards individualised competition periodisation programmes, based on maturation thresholds, for safe and effective long-term athletic development. In this regard, the results highlight promising avenues for future research aimed at the adoption of bioband-based competitions, as proposed by numerous studies in the academic literature [11,12,41].

## Figures and Tables

**Table 1:** Rules differences between the ST and MD1 tournaments.

	ST	MD1
Duration	2 × 25 min	5 × 10 min
Score	Totals goals	Sets won
Substitutions	Free	None (except 5th set)
Special rules		The goalkeeper must play at least one period as an outfield player.
		Direct headers are prohibited.
		The distance between defenders and the offside line is increased for goal kicks.

**Table 2:** Comparison between tournaments for variables linked to impacts.

	ST	MD1	*p*-Value	Effect Size	BF_10_	
	M ± SD [IC 95%]	M ± SD [IC 95%]				
Total impacts (n)	13,922.7 ± 5096 [12,452; 15,296]	15,133.6 ± 4457.2 [13,867; 164,00]	0.082	0.251	0.661	ambiguous
High impacts (n) *	104.5 ± 109.7 [72.5; 134]	86.6 ± 73.1 [65.8; 107]	0.658	0.073	0.304	ambiguous
0–3 g impacts (n)	11,926.1 ± 4272.8 [10,701; 13,084]	13,110.6 ± 3802.9 [12,030; 14,191]	<0.048	0.286	1.002	anecdotal
3–5 g impacts (n) *	1336.5 ± 668.3 [1140; 1514]	1385.6 ± 594.3 [1217; 1555]	0.235	0.194	0.184	ambiguous
5–8 g impacts (n) *	555.6 ± 350.4 [454; 650]	550.8 ± 297.1 [466; 635]	0.996	0.002	0.155	ambiguous
>8 g impacts (n) *	104.5 ± 109.7 [72.5; 134]	86.6 ± 73.1 [65.8; 107]	0.658	0.073	0.304	ambiguous
Total horizontal (n)	16,828.1 ± 7074.9 [14,752; 18,711]	17,552 ± 5961 [15,858; 19,246]	0.388	0.123	0.220	ambiguous
0–3 horizontal (n)	16,286.1 ± 6838 [14,285; 18,109]	17,053 ± 5810 [15,402; 18,705]	0.348	0.134	0.235	ambiguous
3–5 horizontal (n) *	440.8 ± 302.4 [350; 520]	421.2 ± 270.6 [344; 498]	0.499	0.111	0.169	ambiguous
5–8 horizontal (n) *	86.1 ± 91.4 [59; 110]	66.6 ± 65.3 [48; 85.1]	0.313	0.194	0.402	ambiguous
>8 horizontal (n) *	15.2 ± 20.2 [9.29; 20.6]	10.7 ± 14.5 [6.57; 14.8]	0.253	0.194	0.405	ambiguous

*: non normal variables.

**Table 3:** Comparison between tournaments for jump-related variables.

	ST	MD1	*p*-Value	Effect Size	BF_10_	
	M ± SD [IC 95%]	M ± SD [IC 95%]				
Jumps (n) *	8.36 ± 5.59 [6.61; 9.79]	8.24 ± 4.69 [6.91; 9.57]	0.961	0.009	0.156	ambiguous
Steps (n)	6336 ± 2663 [5552; 7043]	6560 ± 2345 [5893; 7226]	0.497	0.097	0.192	ambiguous
Average take off (g) *	2.22 ± 0.73 [2.01; 2.42]	1.77 ± 0.74 [1.56; 1.98]	0.001	0.525	23.97	very strong
Average landing (g) *	4.89 ± 1.01 [4.60; 5.16]	4.21 ± 0.79 [3.98; 4.43]	<0.001	0.583	70.57	very strong
High take off (n) *	1.74 ± 2.17 [1.10; 2.32]	1.12 ± 1.11 [0.82; 1.44]	0.080	0.328	0.776	ambiguous
High landing (n) *	1.72 ± 1.77 [1.19; 2.18]	1.50 ± 1.55 [1.06; 1.94]	0.441	0.143	0.193	ambiguous
3–5 g landing *	6.64 ± 4.67 [5.18; 7.84]	6.74 ± 3.97 [5.61; 7.87]	0.856	0.032	0.156	ambiguous
5–8 g landing *	1.46 ± 1.53 [1; 1.86]	1.24 ± 1.38 [0.85; 1.63]	0.500	0.129	0.200	ambiguous
<8 g landing *	0.260 ± 0.56 [0.09; 0.41]	0.260 ± 0.60 [0.09; 0.43]	0.888	0.004	0.154	ambiguous
0–3 g take off *	6.62 ± 4.26 [5.28; 7.70]	7.12 ± 4.53 [5.83; 8.41]	0.506	0.118	0.201	ambiguous
3–5 g take off *	1.16 ± 1.68 [0.67; 1.61]	0.78 ± 0.84 [0.54; 1.02]	0.186	0.298	0.461	ambiguous
5–8 take off *	0.08 ± 0.27 [0.25; 0.73]	0.04 ± 0.20 [0.12; 0.47]	0.484	0.333	0.210	ambiguous

*: non normal variables.

## Data Availability

Data is contained within the article.
